# Altered Dopamine and Serotonin Metabolism in Motorically Asymptomatic R6/2 Mice

**DOI:** 10.1371/journal.pone.0018336

**Published:** 2011-03-31

**Authors:** Fanny Mochel, Brandon Durant, Alexandra Durr, Raphael Schiffmann

**Affiliations:** 1 INSERM UMR S975, Institut du Cerveau et de la Moelle, Hôpital de La Salpêtrière, Paris, France; 2 AP-HP, Département de Génétique, Hôpital de La Salpêtrière, Paris, France; 3 Université Pierre et Marie Curie, Paris, France; 4 Institute of Metabolic Disease, Baylor Research Institute, Dallas, Texas, United States of America; University of Cambridge, United Kingdom

## Abstract

The pattern of cerebral dopamine (DA) abnormalities in Huntington disease (HD) is complex, as reflected by the variable clinical benefit of both DA antagonists and agonists in treating HD symptoms. In addition, little is known about serotonin metabolism despite the early occurrence of anxiety and depression in HD. *Post-mortem* enzymatic changes are likely to interfere with the *in vivo* profile of biogenic amines. Hence, in order to reliably characterize the regional and chronological profile of brain neurotransmitters in a HD mouse model, we used a microwave fixation system that preserves *in vivo* concentrations of dopaminergic and serotoninergic amines. DA was decreased in the striatum of R6/2 mice at 8 and 12 weeks of age while DA metabolites, 3-methoxytyramine and homovanillic acid, were already significantly reduced in 4-week-old motorically asymptomatic R6/2 mice. In the striatum, hippocampus and frontal cortex of 4, 8 and 12-week-old R6/2 mice, serotonin and its metabolite 5-hydroxyindoleacetic acid were significantly decreased in association with a decreased turnover of serotonin. In addition, automated high-resolution behavioural analyses displayed stress-like behaviours such as jumping and grooming and altered spatial learning in R6/2 mice at age 4 and 6 weeks respectively. Therefore, we describe the earliest alterations of DA and serotonin metabolism in a HD murine model. Our findings likely underpin the neuropsychological symptoms at time of disease onset in HD.

## Introduction

Huntington disease (HD) is an autosomal dominant neurodegenerative disease with complete penetrance. HD is caused by a CAG repeat expansion in the *HTT* gene that encodes huntingtin [1], [2]. Individuals who are at risk can have access to predictive genetic testing in order to determine whether they have inherited the expanded CAG trinucleotide repeat. HD is characterised by progressive motor dysfunction, cognitive decline, and psychiatric disturbance with an age of onset usually between 30 and 50 years old. The concept of “phenoconversion” or “motor onset” does not account for the many individuals who show cognitive or behavioural disturbances several years before the onset of motor symptoms. In particular, anxiety, depression and irritability are prominent symptoms in presymptomatic HD carriers but are too infrequently recognized and therefore undertreated [3], [4].

Dopamine (DA) alterations have been reported in murine models of HD [5] and *post-mortem* tissues from HD patients [6] and may account for both motor and non-motor manifestations of the disease. In particular, DA receptors, i.e. D1 and D2 receptors, and DA uptake sites are reduced in symptomatic HD patients [7], [8] but also in presymptomatic HD carriers [9] suggesting an early dysfunctional DA signalling in HD. Transcriptional deregulation plays an important role in the pathophysiology of HD and the expression of DA receptors is decreased in HD [10]. However, both DA antagonists [11] and agonists [12] have shown some clinical benefit in treating HD symptoms. Schizophrenia-like symptoms can be seen in the early stages of HD and may reflect a hyperdopaminergic state. Similarly, DA depleting treatments such as tetrabenazine, an inhibitor of the vesicular monoamine transporter VMAT-2, improves abnormal movements, i.e. chorea. Although it is possible that some of these apparent contradictory results reflect the dynamic changes that occur in the DA system during the progression of HD, technical bias inherent to the methods of tissue collection may also be at fault. In addition, serotonin (5-HT) metabolism has been little characterized in HD [13], [14]. In particular, *post-mortem* enzymatic changes are likely to interfere with the *in vivo* profile of biogenic amines [15]. In an attempt to circumvent this limitation, and in order to better address the kinetics of DA and serotonin metabolites in R6/2 mice at different stages of the disease, we used a microwave fixation system that instantaneously inactivates brain enzymes while preserving the structure of the brain for regional dissection.

## Materials and Methods

### Mice

All animals were handled in strict accordance with good animal practice as defined by the Texas animal welfare bodies, and all animal work was approved by the institutional animal care and use committee at the Baylor Research Institute, Dallas, TX (#007_001). Four, 8 and 12-week-old transgenic R6/2 mice and wild-type littermates obtained from Jackson Laboratory (Bar Harbor, ME, USA) were maintained on a 12 h lights on 12 h lights off, temperature-controlled environment. Mice were housed 4–5 per cage in an enriched environment. They were given *ad libitum* access to food and water. At two weeks of age tail snips were obtained and sent to Laragen Inc. (Los Angeles, CA), for genotyping and sequencing of CAG repeats. The number of CAG repeats from our R6/2 mouse colony ranged from 106 to 126. Mice were also genotyped for the *Pde6d* gene (Laragen Inc, LA, CA, USA) since *Pde6d* mut/mut is present in about 30% of R6/2 mice bred in a manner where C57BL6CBA is crossed to C57BL6 CBA F1 hybrids. We excluded from the analyses mice that were homozygous for the *Pde6d* mutation since these mice develop blindness overtime [16], representing a confounding factor in neurobehavioural analyses, and in particular for spatial learning tasks.

### Collection of brain samples after microwave fixation

Mice were killed by focused microwave irradiation using a 10 kW Muromachi Microwave Applicator, Model TMW-4012C (Stoelting Co., Wood Dale, IL, USA), as detailed [17]. The system has a specially designed applicator unit that radiates a large amount of microwave energy in a short period of time on a rat or mouse, restrained in the applicator. In addition, the use of a water jacket surrounding the animal holder allows a homogenous repartition of the irradiation on the whole brain surface. Water becomes hot after irradiation and therefore was replaced with new water every time a new mouse was placed. Exposure time, power and position of the holder in the applicator unit were adjusted incrementally to the right settings for each weight/age group using the Muromachi machine. Since exposure time to the focused microwaves was less than 1 second for each animal, there was no concern about animal welfare. Regional dissection was performed on ice in order to isolate striatum, hippocampus and frontal cortex. Following dissection, samples were immediately placed on dry ice and then kept frozen at −80°C until tissue assay.

### Measurement of dopaminergic and serotoninergic amines by HPLC

Tissues were thawed and deproteinized with a volume of perchloric acid (0.4 M) containing DETAPAC (1 mg/ml) and DTE (0.1 mg/ml) such that the dilution factor was 10. After centrifugation, a 5 µL of clear perchloric acid extract was injected directly into the amine HPLC system. DA, 5-HT and their metabolites – 3,4-dihydroxyphenylacetic acid (DOPAC), 3-ortho-methyldopa (3-OMD), 3-methoxytyramine (3-MT), homovanillic acid (HVA) and 5-hydroxyindoleacetic acid (5-HIAA) – were separated on an HPLC column (C18 250×3 mm, 5 µ, Phenomenex, CA); at a flow rate of 0.5 ml/min with a mobile phase consisting of potassium phosphate (0.05 mM), octylsulfate (1 mM) and 14% methanol, adjusted to pH 2.65. Detection was performed using a coulometric electrochemical cell (model 5014B, ESA Inc, Chelmsford, MA) set to a potential of +400 mV. Data were acquired and processed using Coularray for Windows software (ESA Inc, Chelmsford, MA).

### Behavioural studies

#### Home cage

Four digital video cameras were mounted perpendicular to the cages. Video data were analyzed by HomeCageScan software (Clever Systems, Reston, VA, USA). During recording, mice were housed in polycarbonate cages (Lab Product, Seaford, DE, USA) to improve the video tracking of the mice, with minimal bedding (80 ml) to minimize mounding, which can obscure the mouse. Each mouse was recorded individually for 24 h, with 12 h daylight and 12 h dim red light, and then returned to its cage with its littermates. A set of videos were hand scored and compared with HomeCageScan scoring in order to ensure that data encryption was accurate. Among the panel of behaviours that can be analyzed using the HomeCageScan technology [18], hanging, jumping and grooming were selected in view of their relevance to the biochemical profiles that were obtained after microwave fixation. Hanging behaviors were detected when the animal was suspended from the cage top. Grooming was recorded if the animal's body deformed over a defined criteria and over a certain minimum time period, while the animal was situated in the same place. It also required that the animal be in a set of possible postures; likewise, the deformation criteria defined possible shape change patterns over time. Jumping was defined as any movement from a lower position to a higher position and quickly back down to the lower position. It typically required quick elevation of the lower end of the animal above the bedding floor and return back to the bedding floor. Data were expressed as percent of time. We recorded R6/2 and WT mice at 4, 8 and 12 weeks of age.

#### Barnes maze

We used a circular platform (91 cm of diameter) with 20 equally spaced holes (5 cm diameter) along the perimeter and elevated 90 cm above the floor (Stoelting, Wood Dale, IL). Video tracking was performed using TopScan software (Clever Syst, Reston, VA). For stimulus/reinforcement, we used a combination of bright light and 4 equidistant fans blowing air on the table. Visual cues consisted of figures with various shapes and colours. The maze was cleaned out between each mouse using a solution with 1% Incidin solution to avoid olfactory cues. The protocol was adapted from Nature protocols. Briefly, after an adaptation period to the tunnel on day 1, the mice received 2 trials per day with an inter-trial interval of 30 minutes during 4 consecutive days. The probe trials were conducted on day 5 and day 12 in order to test short- and long-term retention. Number of pokes (errors) in each hole and latency to reach the virtual target hole were measured on day 1, 2, 3 and 4. Number of pokes in the target hole (targets) and latency to reach the virtual target hole were measured on day 5 and 12 in order to assess both short- and mid-term retention memory.

### Statistics

For comparison of means and correlations, parametric (ANOVA and Pearson) or non-parametric tests (Spearman) were used as appropriate.

## Results

### Altered DA and serotonin metabolism in R6/2 mice

After verifying that there were no differences between male and female mice for each genotype sacrificed by focused microwave irradiation (data not shown), the values obtained for each metabolite were pooled for both sexes. We found a significant decrease of DA in the striatum of R6/2 mice at 8 and 12 weeks of age, as well as in the hippocampus and frontal cortex of 12-week-old R6/2 mice compared to WT littermates (Table 1). DA metabolites, DOPAC, 3-MT and HVA, were significantly decreased at age 8 and 12 weeks in the striatum, hippocampus and/or frontal cortex of R6/2 mice (Table 1). A significant decrease of striatal DOPAC, 3-MT and HVA was also observed in 4-week-old R6/2 mice (Table 1). In addition, we found significantly reduced 3-MT/DA ratio in the striatum and HVA/DA ratio in the striatum and frontal cortex of R6/2 mice at 4, 8 and 12 weeks of age (Table 1). No overt difference was detected for 3-OMD between R6/2 and WT mice (Table 1).

**Table 1:** Profile of dopamine metabolites in brain of WT and R6/2 mice at 4, 8 and 12 weeks of age.

GENOTYPE	AGE	REGION	N	3-OMD	DA	DOPAC	3-MT	HVA	3MT/DA	HVA/DA
	(weeks)			(nmol/g)	(nmol/g)	(nmol/g)	(nmol/g)	(nmol/g)		
R6/2	4	Striatum	17	0.329±0.058	38.1±3.8	1.97±0.30	0.208±0.043	3.78±0.57	0.0054±0.0009	0.100±0.013
WT			18	0.324±0.048	40.0±5.8	2.49±0.47	0.267±0.053	4.72±1.01	0.0063±0.0014	0.119±0.015
p value				NS	NS	0.002	0.003	0.005	0.021	<0.001
R6/2	4	Hippocampus	17	0.324±0.067	0.728±0.199	0.050±0.042	0.015±0.019	0.224±0.044	0.0183±0.0246	0.346±0.098
WT			18	0.326±0.051	0.777±0.226	0.057±0.039	0.015±0.016	0.270±0.066	0.0168±0.0202	0.355±0.067
p value				NS	NS	NS	NS	0.035	NS	NS
R6/2	4	Frontal Cortex	16	0.321±0.073	4.67±1.43	0.449±0.151	0.034±0.035	1.088±0.320	0.0063±0.0062	0.251±0.062
WT			17	0.310±0.064	4.47±2.47	0.486±0.213	0.025±0.022	1.157±0.361	0.0067±0.0079	0.322±0.122
p value				NS	NS	NS	NS	NS	NS	0.043
R6/2	8	Striatum	15	0.330±0.060	39.3±7.0	1.65±0.25	0.210±0.050	2.60±0.48	0.0055±0.011	0.066±0.009
WT			15	0.310±0.050	59.6±11.2	3.15±0.65	0.450±0.090	5.88±1.21	0.0078±0.0016	0.100±0.017
p value				NS	<0.001	<0.001	<0.001	<0.001	<0.001	<0.001
R6/2	8	Hippocampus	15	0.328±0.058	0.930±0.310	0.209±0.161	0.033±0.009	0.233±0.125	0.0407±0.0198	0.298±0.273
WT			15	0.315±0.048	1.040±0.260	0.217±0.163	0.052±0.020	0.397±0.142	0.0533±0.0256	0.392±0.130
p value				NS	NS	NS	0.004	0.002	NS	NS
R6/2	8	Frontal Cortex	15	0.312±0.065	5.56±2.76	0.564±0.213	0.052±0.014	0.798±0.220	0.0125±0.0093	0.170±0.072
WT			14	0.304±0.036	7.17±2.65	0.808±0.263	0.085±0.031	1.634±0.468	0.0147±0.0127	0.260±0.120
p value				NS	NS	0.011	0.002	<0.001	NS	0.02
R6/2	12	Striatum	18	0.275±0.072	25.9±5.2	0.76±0.29	0.156±0.036	1.77±0.42	0.0061±0.0013	0.068±0.008
WT			15	0.230±0.030	55.0±11.1	2.39±0.97	0.390±0.080	4.95±1.06	0.0072±0.0010	0.091±0.017
p value				0.025	<0.001	<0.001	<0.001	<0.001	0.007	<0.001
R6/2	12	Hippocampus	18	0.237±0.051	0.710±0.230	0.108±0.059	0.029±0.015	0.223±0.093	0.0473±0.0357	0.337±0.176
WT			16	0.225±0.045	1.090±0.540	0.132±0.071	0.050±0.024	0.428±0.305	0.0562±0.0369	0.420±0.249
P value				NS	0.009	NS	0.004	0.01	NS	NS
R6/2	12	Frontal Cortex	18	0.286±0.259	3.98±1.50	0.366±0.181	0.055±0.020	0.605±0.084	0.0169±0.0143	0.187±0.096
WT			15	0.207±0.034	5.15±1.51	0.744±0.236	0.071±0.029	1.243±0.253	0.0147±0.0077	0.259±0.088
p value				NS	0.021	<0.001	0.068	<0.001	NS	0.033

NS: non significant.

Furthermore, in all 3 regions analyzed – striatum, hippocampus and frontal cortex – we found significantly decreased 5-HT and 5-HIAA in R6/2 mice at 4, 8 and 12 weeks of age compared to WT littermates (Table 2). The ratio of 5-HIAA to 5-HT was also quite significantly decreased in the striatum, hippocampus and frontal cortex of 4, 8 and 12-week-old R6/2 mice (Table 2) suggesting reduced serotonin turnover in HD mice [19]. We did not find any correlation between the number of CAG repeats and serotoninergic or dopaminergic amines.

**Table 2:** Profile of serotonin metabolites in brain of WT and R6/2 mice at 4, 8 and 12 weeks of age.

GENOTYPE	Age	REGION	N	5-HT	5-HIAA	5-HT/5-HIAA
	(weeks)			(nmol/g)	(nmol/g)	
R6/2	4	Striatum	17	6.14±1.19**	1.27±0.36**	0.206±0.042**
WT			18	7.34±1.14	2.26±0.35	0.311±0.045
R6/2	4	Hippocampus	17	6.12±0.87**	1.26±0.31**	0.206±0.044**
WT			18	8.38±1.32	2.47±0.41	0.300±0.059
R6/2	4	Frontal Cortex	16	6.57±1.24**	0.65±0.23**	0.099±0.024**
WT			17	8.51±0.86	1.13±0.17	0.133±0.019
R6/2	8	Striatum	15	5.67±0.79**	0.48±0.11**	0.085±0.017**
WT			15	10.16±1.73	2.61±0.56	0.259±0.045
R6/2	8	Hippocampus	15	5.23±0.71**	0.45±0.12**	0.085±0.020**
WT			15	11.88±2.10	3.03±0.72	0.257±0.048
R6/2	8	Frontal Cortex	15	6.75±1.19**	0.22±0.05**	0.033±0.005**
WT			14	12.32±1.69	1.33±0.23	0.110±0.027
R6/2	12	Striatum	18	4.85±1.30**	0.39±0.12**	0.082±0.015**
WT			15	9.14±2.47	2.46±0.60	0.275±0.046
R6/2	12	Hippocampus	18	5.40±1.11**	0.44±0.16**	0.082±0.023**
WT			16	12.02±2.27	3.30±0.77	0.278±0.055
R6/2	12	Frontal Cortex	18	6.86±1.19**	0.27±0.18**	0.041±0.028**
WT			15	11.86±2.01	1.49±0.24	0.128±0.025

**p<0.001 compared to WT mice.

Of note, compared to mice that were sacrificed using CO2 asphyxiation, mice that were sacrificed by focused microwave irradiation displayed significantly lower striatal levels of dopaminergic metabolites, and especially a 4-fold difference in DOPAC and a 15-fold difference in 3-MT, but a 2-fold increase in 5-HT in the striatum (data not shown).

### Increased stress-like behaviours and altered spatial learning in R6/2 mice

#### Home Cage

**Figure 1:**
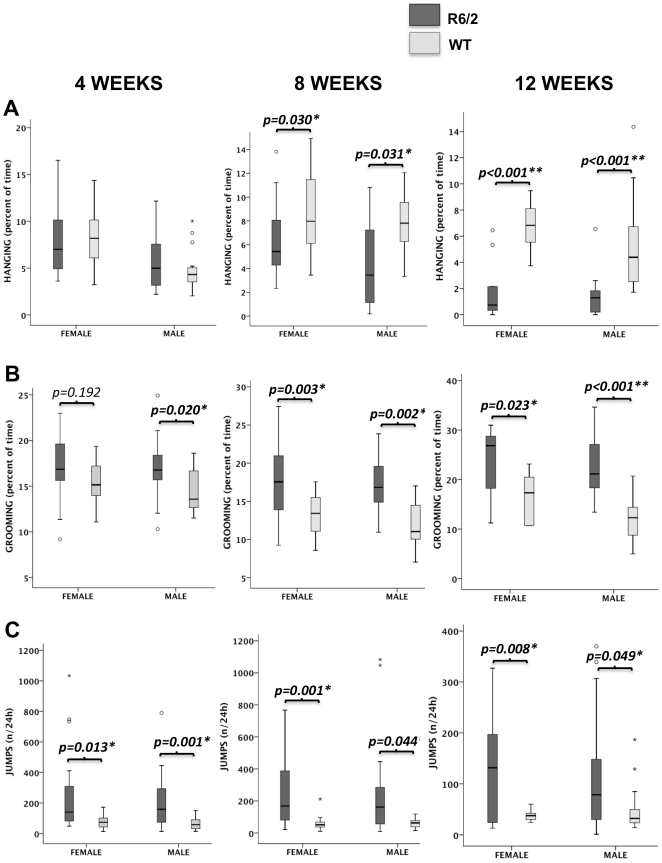
HomeCageScan behaviours in R6/2 mice at age 4, 8 and 12 weeks. (A) Total time spent hanging, (B) grooming and (C) number of jumps, from the HomeCage recording. n = 20 animals/group.

We did not find any difference between males and females for the different phenotypes of interest (Figure 1). However, the parameters were analyzed separately for male and female since body weight can be a confounding factor for motor functions in mice. We found a significant decrease of total time spent hanging in 8 and 12-week-old R6/2 males and females compared to WT (Figure 1A). These decrements were similar in both light and dark phases (data not shown). However, we did not observe any abnormality in hanging (Figure 1A), or other locomotor phenotype, in HD mice at 4 weeks of age indicating that the mice were motorically asymptomatic at this age. Importantly, repetitive behaviours recognized as stress-like behaviours [20], [21] were already present at 4 weeks of age with significantly increased grooming behaviours in R6/2 males (Figure 1B) and jumps in both R6/2 males and females (Figure 1C). At age 8 and 12 weeks, grooming and jumping behaviours remained significantly increased in R6/2 males and females (Figures 1B and 1C).

#### Barnes maze

**Figure 2:**
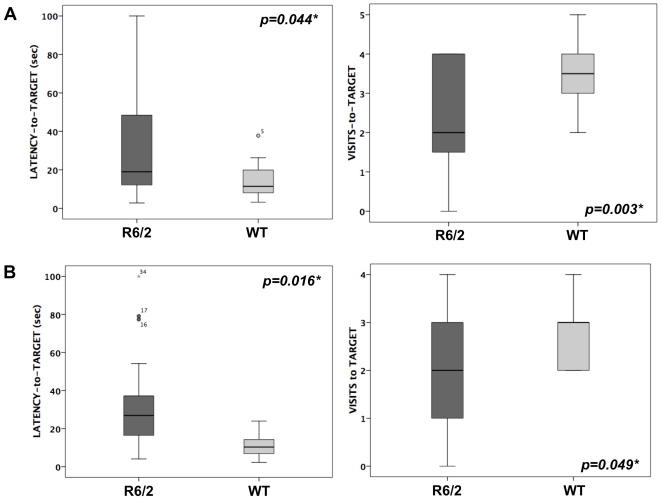
Barnes maze test in 6-week-old R6/2 and WT mice. Latency to reach the virtually target hole and numbers of pokes in the target hole were measured on day 5 (A) and day 12 (B). n = 20 animals/group.

The Barnes maze test was performed at 6 weeks of age, i.e. at a presymptomatic age for motor phenotype in R6/2 mice. After verifying that there were no differences between male and female mice for each genotype (data not shown), the values obtained for each parameter of the Barnes maze test were pooled for both sexes. We found significantly increased latency to reach the target hole at day 1 in R6/2 mice compared to WT littermates (t = 81±75 sec vs 28±20 sec, p = 0.013) as well as day 2 (t = 52±39 sec vs 23±14 sec, p = 0.010), day 3 (t = 44±34 sec vs 20±13, p = 0.017) and day 4 (t = 32±20 sec vs 18±10 sec, p = 0.019). R6/2 mice also made significantly more errors at day 1 (n = 10±7 vs 5±4, p = 0.046) and day 3 (n = 9±4 vs 6±3, p = 0.010) compared to WT mice. At day 5 and day 12, the latency to reach the target hole was significantly increased in the R6/2 mice while the number of targets was significantly lower (Figure 2), indicating altered short- and mid-term retention memory in HD mice.

## Discussion

Using a microwave fixation system, we found a significant decrease of DA, 3-MT and HVA in the striatum of R6/2 mice at 8 and 12 weeks of age paralleling motor deficit in these mice. Some of the dopaminergic alterations were also seen in non-striatal regions such as hippocampus and frontal cortex, underlining the early aberrant connectivity of dysfunctional frontostriatal circuits in HD [22]. Likewise, several studies reported decreased CSF levels of HVA in HD patients prior to the *HTT* gene identification [23], [24], [25]. Importantly, we showed for the first time that the DA loss was preceded by altered DA metabolism, i.e. decreased 3-MT and HVA levels, in striatum and frontal cortex of 4-week-old motorically asymptomatic R6/2 mice. Indeed, the use of a video-based behavior-recognition technology allowed us to confirm that mice sacrificed at 4 weeks of age were at the presymptomatic motor stages of the disease as previously shown [18], [26]. Radiolabeled studies from synaptosomes showed normal DA release and uptake in R6/2 mice [27], but direct measurements of DA neurotransmission using fast-scan cyclic voltammetry at carbon-fiber microelectrodes showed that DA release was severely compromised in brain slices from 12-week-old R6/2 mice [28]. While some studies hypothesized a deficiency of DA exocytosis due to impaired vesicular packaging [28], others indicated a decline in the number of DA reserve pool vesicles available for mobilization [29]. Since the injection of 3-nitropropionate (3NP) – an inhibitor of the mitochondrial enzyme succinate dehydrogenase – in WT mice reproduced the same reduction of DA release, the altered vesicular packaging was thought to be an ATP-dependant process [28]. Similarly, progressive declines in striatal D2-receptor binding were correlated with concurrent hypometabolism in presymptomatic individuals [30]. While our study does not directly assess dopamine release, the decreased 3-MT/DA and HVA/DA ratio in the context of decreased DA levels [31] at 8 and 12 weeks are consistent with more direct measurements of reduced dopamine release at the same time points [5], [29].

Moreover, we demonstrated for the first time that serotonin metabolism is impaired at the earliest stages in the R6/2 transgenic mouse model of HD. We found that both 5-HT and 5-HIAA were significantly decreased in the striatum, hippocampus and frontal cortex of 8 and 12-week-old R6/2 mice. Unlike previous studies that used common sacrificial methods [13], our study revealed a significant decrease of both serotoninergic metabolites as early as age 4 weeks in the 3 brain regions that were analyzed in our HD mice. Serotonin turnover rate, as suggested by the 5-HT to 5-HIAA ratio, was also likely altered in R6/2 mice compared to WT littermates starting at 4 weeks of age. Reduced expression of specific serotonin receptors and serotonin transporter, as recently reported in pre-motor symptomatic R6/1 mice, may explain this abnormal serotoninergic pattern [32]. Of note, treating 6-week-old R6/2 mice with sertraline – a selective serotonin reuptake inhibitors (SSRI) antidepressant – led to improved survival, motor performance and neurogenesis [33]. The same therapeutic benefits were reported when treating N171-82Q mice – another transgenic mouse model of HD – with the SSRI sertraline [34].

The use of an advanced automated video tracking system also allowed us to identify early behavioural abnormalities in R6/2 mice with increased grooming and jumping at 4 weeks of age. Jumping was previously shown to decrease in R6/2 mice [18] and was thought to reflect the locomotor decline in HD mice. By contract, and despite evidence of motor symptoms, as shown at age 8 and 12 weeks by significantly decreased hanging, our data displayed a clear increase of behaviours such as jumping and grooming. These repetitive behaviours have been interpreted to indicate stress [20], [21]. Thus, this neuropsychological profile may be a behavioural correlate of the early serotonin alterations that we found in R6/2 mice as early as 4 weeks of age. This would warrant further trials with pharmacological agents altering either serotonin or dopamine pathways in HD preclinical models in order to better delineate the link with the early stress-like behaviors that we observed. Of note, our observation of serotonin depletion in 4-week-old mice goes along with the efficacy of SSRI antidepressant drugs or even atypical neuroleptics such as olanzapine – i.e. partial agonists of 5-HT1 receptors – in treating anxiety and depression at the early stage of HD. Accordingly, their efficacy is superior compared to benzodiazepines usually proposed for treatment of anxiety (A. Durr, personal observation). We also found altered cognition with decreased spatial learning in 6-week-old R6/2 mice. Considering that serotonin and DA neurotransmitters play a key role in modulating synaptic transmission in the brain [35], the combined deficit of both serotonin turnover and DA release likely underlies the learning and memory deficits that we observed. Of note, none of the serotonin and DA metabolites correlated with the number of CAG repeats. This may reflect a threshold effect in which the magnitude of changes between R6/2 and WT mice – up to 2-fold difference for some neurotransmitters – would surpass the specific effect of CAG repeats. The lack of correlation may also be explained by the narrow range of CAG repeats in our HD mouse colony.

This is the first study reporting the levels of DA and serotonin metabolites in different brain regions of WT mice after microwave fixation. The instantaneous inactivation of brain enzymes by the microwave prevents *post-mortem* changes in particular for high-energy phosphates [36] and phosphorylation reactions [17]. Here we found that dopaminergic and serotoninergic neurotransmitters were also sensitive to microwave fixation, and in particular DOPAC, 3-MT and 5-HT [13]. Consequently, we were able to detect significant changes in both DA and serotonin metabolism at very early stages of HD pathology in R6/2 mice that were not displayed using other sacrificial methods. These abnormal neurotransmitters profiles mirrored the presence of increased stress-like behaviours in otherwise motorically asymptomatic 4-week-old HD mice. Altogether, this study underscores that neurotransmitters alterations occur early in HD pathogenesis and that neuropsychological symptoms should be investigated and treated at the presymptomatic stages of the disease.
